# LncRNA HOTAIR/miR-9-5p/FOXP1 axis modulates cerebral ischemia-reperfusion injury via NLRP3 inflammasome activation

**DOI:** 10.1016/j.ibneur.2026.08.005

**Published:** 2026-08-11

**Authors:** Tingting Liu, Xiaosong Zhu, Fengjuan Zhuo, Shanxin Peng

**Affiliations:** a Department of Vasculocardiology, Linyi People’s Hospital, Shandong Second Medical University, Linyi, Shandong 276000, PR China; b Department of Infection Management, Linyi People’s Hospital, Shandong Second Medical University, Linyi, Shandong 276000, PR China; c Department of Clinical Microbiology, Linyi People’s Hospital, Shandong Second Medical University, Linyi, Shandong 276000, PR China

**Keywords:** Cerebral ischemia-reperfusion injury, HOTAIR, MiR-9-5p, NLRP3

## Abstract

**Objective:**

This study aimed to investigate the association between the HOTAIR-miR-9–5p axis and the inflammatory response in ischemic stroke (IS) and elucidate the underlying molecular mechanisms.

**Methods:**

Middle cerebral artery occlusion/reperfusion (MCAO/R) and oxygen-glucose deprivation/reoxygenation (OGD/R) were applied to simulate ischemic/reperfusion conditions *in vivo* and *in vitro*. The expression levels of HOTAIR and miR-9–5p in the serum of patients or in the brain tissue of MCAO/R mice were assessed by qRT-PCR, and the secretion levels of IL-1β and IL-18 were analyzed by ELISA. Dual-luciferase reporter assays, RNA-binding protein immunoprecipitation (RIP), and RNA pull-down assays were performed to validate the target relationship. Western blotting was applied to assess the expression of NLRP3, CASP1, and FOXP1. Moreover, MCAO/R mice with intracerebroventricular injection of antagomir-9–5p were used to evaluate the effect of antagomir-9–5p on cerebral ischemia-reperfusion injury (CIRI).

**Results:**

A significant association was observed between the HOTAIR-miR-9–5p axis and inflammation in both IS patients and MCAO/R mice. HOTAIR was abnormally expressed at a low level, whereas miR-9–5p and the associated protein NLRP3 inflammatory response were increased in the serum of IS patients, as well as in the brain tissue of MCAO/R mice and in SH-SY5Y cells. Mechanistically, miR-9–5p negatively regulated the expression of HOTAIR and FOXP1. Furthermore, HOTAIR was found to regulate NLRP3 expression via the miR-9–5p/FOXP1 pathway. Functional experiments revealed that silencing miR-9–5p protected against cerebral ischemia/reperfusion injury and suppressed NLRP3 inflammasome activation.

**Conclusion:**

These findings collectively demonstrate that the HOTAIR/miR-9–5p/FOXP1 axis plays a critical role in NLRP3 inflammasome activation following IS, suggesting that its blockade could be a potential therapeutic strategy for ischemic brain injury.

## 1 Introduction

Stroke is one of the leading causes of devastating clinical conditions, leading to death and permanent disability (Vinding et al., 2023). Ischemic stroke (IS), which has high mortality and morbidity rates, accounts for approximately 85% of all stroke cases. Ischemic stroke is characterized by a sudden interruption of cerebral blood flow in a specific brain region (Palomino-Antolin et al., 2022). Endovascular thrombectomy or drug thrombolysis is the most effective treatment for acute ischemic stroke, restoring cerebral blood oxygenation (Mendelson and Prabhakaran, 2021). However, reperfusion may trigger cerebral ischemia-reperfusion injury (CIRI), posing an additional treatment challenge (Lim et al., 2021). CIRI involves hypoxia, oxidative stress, and inflammation, leading to necrosis, apoptosis, and autophagy in ischemic brain tissue (Meng et al., 2023). Thus, identifying effective CIRI therapeutic targets and mechanisms is crucial.

Ischemic stroke triggers sterile inflammation, with early microglial activation and cytokine release, which is key to cell death and brain injury (Yao et al., 2022). It is now understood that NLRP3 inflammasome activation drives caspase−1-mediated maturation and release of IL-1β and IL-18, exacerbating neuroinflammation and secondary brain injury (Lin et al., 2025). Growing evidence suggests that the activation of the NLRP3 inflammasome plays a significant role in the pathogenesis of ischemic stroke (Palomino-Antolin et al., 2022, Lin et al., 2025, Bellut et al., 2021). Pharmacological/genetic NLRP3 inhibition reduces endothelial hypoxic injury, maintains blood-brain barrier (BBB) integrity, and improves neurological outcomes in preclinical research (Bellut et al., 2021). Regulation of NLRP3 inflammasome activation may contribute to the prognosis of IS (Palomino-Antolin et al., 2022). Accordingly, identifying effective therapeutic targets and elucidating the underlying mechanisms of CIRI is crucial.

Long non-coding RNAs (lncRNAs), a family of RNA molecules exceeding 200 nucleotides in length and lacking the ability to encode proteins, play pivotal roles in regulating various biological processes, including chromatin modification, transcriptional regulation, and intracellular transport (Liu et al., 2021, Bridges et al., 2021). Accumulating evidence indicates that aberrant expression of lncRNAs, such as THAP7-AS1, NEXN-AS1, and PART1 (Pan et al., 2025, Wang et al., 2025, Xu et al., 2025), in IS contributes to its pathogenesis. Homeobox antisense non-coding RNA (HOTAIR) has been identified as a potential therapeutic target in diverse cancers (Amicone et al., 2023); however, reports on its involvement in ischemic stroke remain scarce (Wang et al., 2022). Nevertheless, the precise role of HOTAIR in activating the NLRP3 inflammasome during IS, and the underlying molecular mechanisms, have not been fully elucidated. Furthermore, microRNAs (miRNAs) function as post-transcriptional regulators of gene expression by binding to the 3’ untranslated regions (UTRs) of target messenger RNAs (mRNAs) (Li et al., 2025). Elevated levels of miR−9–5p have been reported in stroke, contributing to brain damage following IS (Bache et al., 2020, Yan et al., 2020). However, whether miR−9–5p participates in NLRP3 inflammasome activation in IS remains unknown.

This study sought to elucidate the role of the HOTAIR/miR−9–5p/FOXP1 axis in IS progression. We hypothesized that inhibiting miR−9–5p may suppress NLRP3 inflammasome activation after stroke. The present research investigated the role of the HOTAIR/miR−9–5p axis in CIRI and its possible underlying molecular mechanisms.

## 2 Methods

### 2.1 Ethics statement

All experiments were approved by the Science and Technology Ethics Committee of Linyi People's Hospital of Shandong Second Medical University (approval No.202411-H−057). The study also conformed to the Declaration of Helsinki. Prior to conducting this study, written informed consent was obtained from all participants. The animal experiments were conducted with the approval of the Animal Ethics Committee of Linyi People's Hospital (approval No.202411-A−005) and in strict accordance with the recommendations of the Guide for the Care and Use of Laboratory Animals, published by the US National Institutes of Health. Adequate measures were taken to minimize animal pain and distress.

### 2.2 Clinical samples and collection

Patient recruitment was conducted from November 2024 to April 2025. A total of 24 patients diagnosed with acute ischemic stroke (AIS) and 24 healthy individuals from the Linyi People’s Hospital were recruited in this study.

Inclusion criteria: patients who met the diagnostic criteria for ischemic stroke established by the Fourth National Conference on Cerebrovascular Diseases. All patients were confirmed by CT or magnetic resonance imaging and were admitted to the hospital within 72 h of onset.

Exclusion criteria: acute infection, tumor or autoimmune disease, acute coronary syndrome, valvular disease and atrial fibrillation, arteritis and peripheral vascular disease, hepatorenal insufficiency and severe cardiac insufficiency, hematological disease, and recent surgery or trauma.

Blood samples were collected from all participants into tubes containing a separation gel. Serum was separated from the blood and partitioned into aliquots. One portion was processed to detect the relative expression levels of lncRNA HOTAIR and miR−9–5p via qRT-PCR. The remaining portion was used to quantify cytokine levels using ELISA.

### 2.3 MCAO/R Mice Model and Treatment Groups

The design and reporting of all animal experiments adhered to the ARRIVE guidelines 2.0 for animal research, and all animal experiments were initiated in April 2025. Male C57BL/6 mice (specific pathogen-free, 6–8 weeks old, 18–22 g) were obtained from Charles River Laboratories (Beijing, China), and a total of 42 mice were included in this study. The animals were acclimated to a controlled temperature of 20 ± 2°C and a 12 h light/dark cycle, with free access to food and water. Mice were randomly assigned to the Sham group and MCAO/R group using a computer-generated random number sequence.

The MCAO method was employed to induce focal cerebral ischemia in all experimental groups, except the Sham cohort, as previously described (Li et al., 2026). Briefly, anesthesia was maintained via isoflurane inhalation (3% for induction, 1.5% for maintenance). A midline skin incision was made to expose the left common carotid artery, external carotid artery (ECA), and internal carotid artery (ICA). Subsequently, a silicone-coated 6–0 nylon monofilament with a rounded tip was introduced into the left ICA via the ECA stump to occlude the origin of the middle cerebral artery (MCA). The intraluminal suture was left in place for 60 min to induce ischemia, after which it was withdrawn to allow reperfusion. The SHAM group (n = 6) received identical surgical treatment, except for the omission of filament insertion.

Inclusion criteria of MCAO/R mice: (1) A reduction in regional cerebral blood flow (rCBF) of ≥ 70% (or to ≤ 20% of baseline) during the insertion of the filament, monitored by Laser Doppler flowmetry (LDF). (2) A modified Neurological Severity Score (mNSS) ≥ 2. (3) Successful survival until the predetermined experimental endpoint.

Exclusion criteria of MCAO/R mice: (1) Intraoperative or postoperative complications, including massive subarachnoid hemorrhage confirmed by necropsy, or premature death before the endpoint. (2) Severe weight loss or inability to eat/drink independently requiring humane intervention before the endpoint. No animals or data points were excluded from any group. Each animal was treated as an independent statistical unit, and its data were reported individually.

Neurological function was scored at 6, 24, and 72 h following MCAO/R. At each time point, blood was collected from a cohort of 6 mice, of which 3 were then euthanized for subsequent experiments. Mice were euthanized by an overdose of pentobarbital sodium (120 mg/kg, i.p.), and then transcardial perfusion with saline was performed prior to brain extraction.

To study the mechanistic role of miR−9–5p in MCAO/R mice, the mice were randomly assigned to the following groups (n = 6 per group; total: 18 mice): (1) Sham group, (2) MCAO/R + NC group, (3) MCAO/R + Antagomir−9–5p group. Antagomir−9–5p or antagomir NC was administered via a left intracerebroventricular injection, with the groups divided accordingly.

Six mice in one group served as one experimental unit, consistent with a previous study (Guo et al., 2025). The group sizes were determined to achieve adequate statistical power to detect biologically relevant effects, while yielding sufficient brain tissue for concurrent histological sectioning and molecular analyses. To minimize confounding factors, animals were randomly assigned to groups with surgeries performed in a randomized order. All treatments and measurements were conducted in a blinded manner. Cage locations were rotated to prevent environmental bias, and behavioral/biochemical assays were performed at consistent times to reduce circadian effects.

### 2.4 Infarct volume measurement and assessment of neurological deficits

The infarct volume was determined in brain sections using a 1.5% TTC staining protocol established in prior reports (Li et al., 2026). Individual brain slices were imaged, and the infarct area was quantified using ImageJ software.

To dynamically assess neurological deficits, the mNSS, which includes motor, reflex, and balance examinations, was performed on days 0, 1, and 3 post-reperfusion, as previously described (Ge et al., 2024). The scoring system ranges from 0 to 14 points; a higher score correlates with greater severity of neurological injury.

### 2.5 Cell culture and oxygen-glucose deprivation/reoxygenation (OGD/R) administration

Human neuroblastoma cell line SH-SY5Y (ATCC) was maintained in a 1:1 ratio of DMEM and Ham’s F12 medium (DMEM/F12), supplemented with 10% FBS, 100 U/mL penicillin, and 100 mg/mL streptomycin. Cells were incubated in a standard incubator at 37° C with 5% CO_2_.

To induce OGD/R injury, SH-SY5Y cells were maintained in glucose-free medium within a hypoxic chamber (95% N_2_, 5% CO_2_) for 4 h. After the insult, the medium was replaced with normal growth medium supplemented with 4.5 g/L glucose and 10% FBS, and the cells were allowed to recover overnight at 37° C in a 5% CO_2_ incubator (Feng and Liu, 2025).

### 2.6 Cell transfection

HOTAIR overexpression plasmid (pcDNA3.1-HOTAIR), FOXP1 siRNA (si-FOXP1), miR−9–5p mimics (agomir−9–5p), miR−9–5p inhibitor (antagomir−9–5p), and corresponding negative controls (pc-NC, si-NC, agoNC, and antaNC) were all obtained from Riobio Inc. (Guangzhou, China). According to the manufacturer’s instructions, they were transfected into SH-SY5Y cells using Lipofectamine 2000 (Invitrogen, Carlsbad, CA, USA). Cells and cell supernatant were collected for the subsequent cell experiments. Each treatment was performed at least three times.

### 2.7 Quantitative real-time PCR

Total RNAs were extracted from the serum of participants, SH-SY5Y cells or peri-infarction cortex tissue of mice using RNAzol reagent (Invitrogen, Shanghai, China). The qRT-PCR of miRNA and lncRNA was performed using MiDETECT A Track miRNA qRT-PCR kits (Riobio Inc., Guangzhou, China) and riboSCRIPT mRNA/lncRNA qRT-PCR Starter Kit (Riobio Inc., Guangzhou, China), respectively. Quantitative real-time PCR (qRT-PCR) was performed to measure the relative expression levels of NLRP3, FoxP1, caspase−1, and interleukin−1β (IL-1β). Oligonucleotide primers were synthesized by BioSune Biotechnology (Shanghai, China) and are listed in Table 1. cDNA synthesis was performed using the PrimeScript RT Reagent Kit (Takara Bio, Dalian, China), and real-time amplification was executed using SYBR Premix Ex Taq (Takara Bio) according to the manufacturer's instructions. ABI7500 systems (Applied Biosystems, CA, USA) were used for all qRT-PCR. Relative quantification was conducted by using the 2^-ΔΔCt^ method.

**Table 1:** Primer sequences.

Name of primer	Sequences (5′→3′)
GAPDH-m	F: AGGTCGGTGTGAACGGATTTG
	R: TGTAGACCATGTAGTTGAGGTCA
NLRP3-m	F: TCCTGGTGACTTTGTATATGCGT
	R: TTCTCGGGCGGGTAATCTTC
FOXP1-m	F: CATGCCTCTACCAATGGACAGC
	R: GAAGTCGTCACAAACCGCCTCA
CASP1-m	F: CCCAAGCTTGAAAGACAAGCC
	R: CCTTGTTTCTCTCCACGGCAT
GAPDH-h	F: GCTCTCTGCTCCTCCTGTTC
	R: GTTGACTCCGACCTTCACCT
IL-1β-h	F: ATGATGGCTTATTACAGTGGCAA
	R: GTCGGAGATTCGTAGCTGGA
NLRP3-h	F: GATCTTCGCTGCGATCAACAG
	R: CGTGCATTATCTGAACCCCAC
CASP1-h	F: TTTCCGCAAGGTTCGATTTTCA
	R: GGCATCTGCGCTCTACCATC

### 2.8 Enzyme-linked immunosorbent assay (ELISA) detection

The serum levels of IL-1β and IL-18 were measured using a commercial ELISA kit (MultiSciences, Hangzhou, China) according to the manufacturer's instructions.

### 2.9 Reporter vectors construction and luciferase reporter assay

The 3′UTRs of HOTAIR or FOXP1, containing the predicted miR−9–5p binding site, were amplified by PCR and cloned into the pGL3 luciferase reporter vector to generate the LUC-HOTAIR-wt or LUC-FOXP1-wt vectors. Mutations at the seed pairing region were induced via site-directed mutagenesis.

Luciferase reporter assay was performed using a Dual Luciferase Assay System Kit (Promega, Madison, WI, USA) following the manufacturer’s protocols. SH-SY5Y cells were co-transfected with a firefly luciferase reporter plasmid (wt or mut), pRL-TK (as an internal control), and the corresponding miRNA mimic or a randomized oligonucleotide as a control. At 48 h post-transfection, the relative luciferase activities were measured using a dual-luciferase assay kit (Promega) and calculated by normalizing firefly luciferase activity against that of Renilla. Each set of assays was performed in triplicate.

### 2.10 RNA pull-down assay

The RNA extracts from SH-SY5Y samples were incubated with the biotin-labelled miR−9–5p probe (Bio-miR−9), its corresponding mutated oligos (Bio-miR−9-Mut), or a biotin-labelled non-specific control RNA as Bio-NC, along with the magnetic beads for 4 h. The pulled-down complex was monitored by qRT-PCR.

### 2.11 RNA immunoprecipitation (RIP) assay

SH-SY5Y cells were lysed and incubated overnight at 4°C with magnetic beads coupled to Argonaute2 (Ago2) antibody (Abcam, Cambridge, UK) or Immunoglobulin G (IgG) antibodies. The enrichment levels of HOTAIR, miR−9–5p, and FOXP1 were subsequently determined by qRT-PCR.

### 2.12 Western blotting

Protein from SH-SY5Y cells or brain tissues was extracted using RIPA lysis buffer (Beyotime, Nantong, China). Protein concentration was determined using a BCA kit (Solarbio Beijing, China). Equal amounts of lysate from each sample were separated by 10% SDS-PAGE and transferred to PVDF membranes (Millipore, Billerica, USA), followed by blocking with 5% nonfat milk for 2 h at room temperature. Subsequently, the membranes were incubated with primary antibodies at 4 °C overnight: NLRP3 (Abcam ab263899; 1:1000), FOXP1 (Abcam ab32010; 1:500), caspase−1 (Servicebio 87271–4-RR; 1:1000), IL-1β (Abcam abab28318; 1:1000) and GAPDH (Abcam ab181602; 1:5000) as a control. After three washes, the membranes were further incubated with HRP-conjugated secondary antibodies for 30 min at room temperature (25 °C), then washed three times with TBST (5 min each). After ECL (Millipore WBKLS0500) exposure, the films were scanned, and the intensity values of each band were determined using an Alphalmager HP system (Cell Biosciences Inc., Santa Clara, CA, USA). The Prestained Protein Marker II (10–200 kDa; Servicebio, G2058–250UL) served as the molecular weight standard for Western blotting.

### 2.13 Statistical analysis

Normality testing confirmed that all datasets followed a normal distribution. All results were statistically analyzed using Student’s *t*-test or one-way analysis of variance using SPSS 27.0 (IBM, Illinois, USA) and GraphPad Prism 5 (GraphPad, CA, USA). The experimental data were expressed as the mean± standard deviation (SD) from three independent experiments. Statistical significance was set at *P* < 0.05.

## 3 Results

### 3.1 Association of the HOTAIR-miR−9–5p axis with the inflammatory response in IS patients

**Fig. 1:**
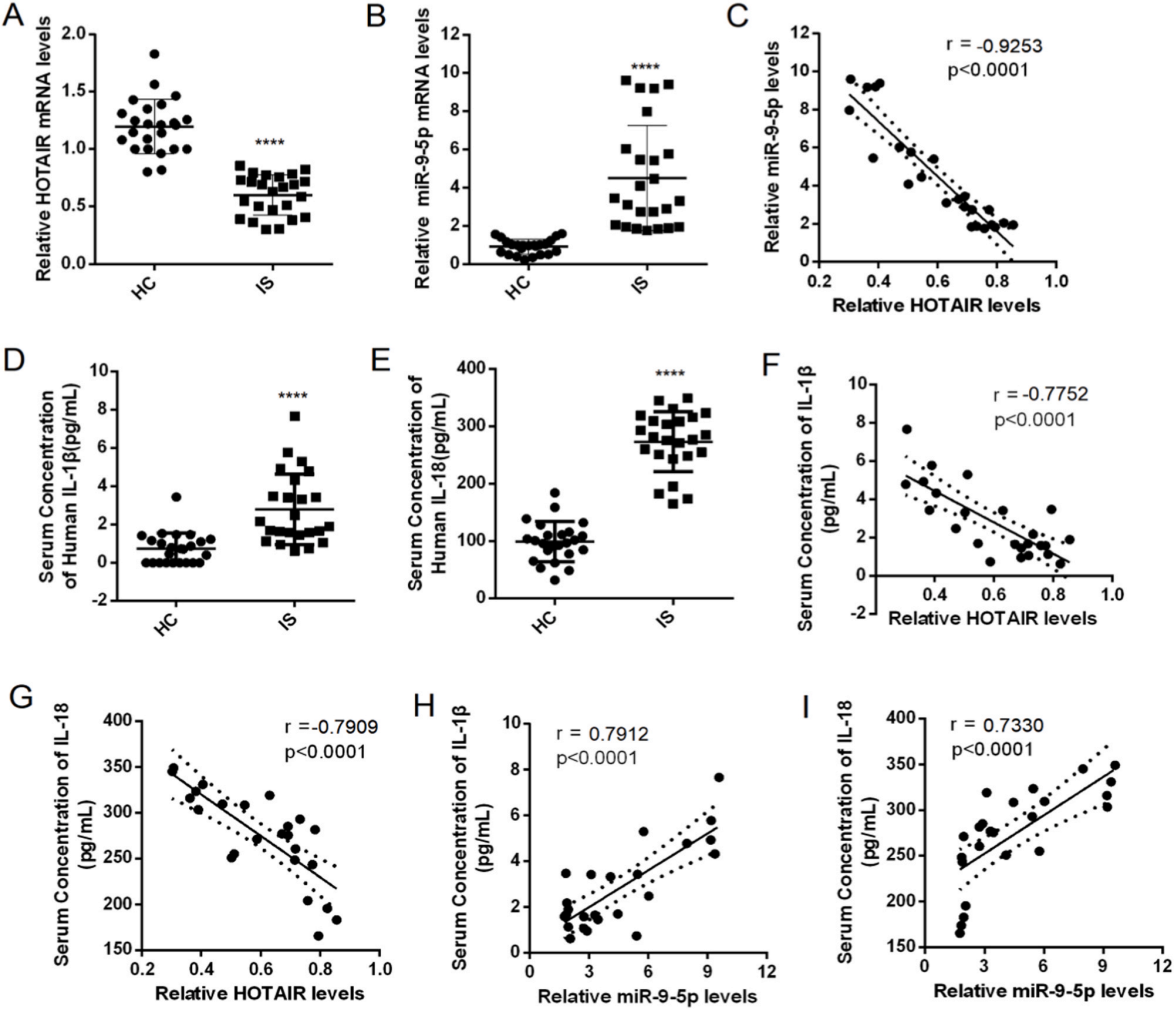
Clinical significance of the HOTAIR-miR−9–5p axis and its association with the inflammatory response in ischemic stroke (IS) patients. (A-B) The serum levels of HOTAIR and miR−9–5p were measured in patients with ischemic stroke (IS) and healthy controls (HCs). (C) Pearson correlation analysis was conducted to examine the expression relationship between HOTAIR and miR−9–5p in the serum of IS patients. (D-E) The serum concentrations of IL-1β and IL-18 in IS patients and HCs were quantified using ELISA. (F-I) Pearson correlation analysis was performed to evaluate the associations between HOTAIR/miR−9–5p levels and IL-1β/IL-18 levels in the serum of IS patients. *r* represents the correlation coefficient. *N* = 24 per group. Data are expressed as mean ± standard deviation (SD). ****P < 0.0001 compared to HC.

Blood samples from IS patients were collected within 6 h of stroke onset. qRT-PCR revealed low-level expression of HOTAIR in the serum of IS patients compared with the healthy control (HC) group (Fig. 1A). However, miR−9–5p levels were significantly increased in the serum of IS patients (Fig. 1B). Furthermore, correlation analysis revealed that serum HOTAIR levels were negatively correlated with miR−9–5p levels (rs = −0.9223, *P* < 0.001) (Fig. 1C).

In the IS group, serum IL-1β and IL-18 levels were significantly elevated (Fig. 1D-E). Pearson’s correlation analysis demonstrated that the expression levels of IL-1β and IL-18 were positively correlated with miR−9–5p expression in serum but negatively correlated with HOTAIR expression (Fig. 1F-I). These results collectively reveal that the HOTAIR-miR−9–5p axis may contribute to the inflammation response in IS patients.

### 3.2 Association of the HOTAIR-miR−9–5p axis with the inflammatory response in MCAO/R mice

**Fig. 2:**
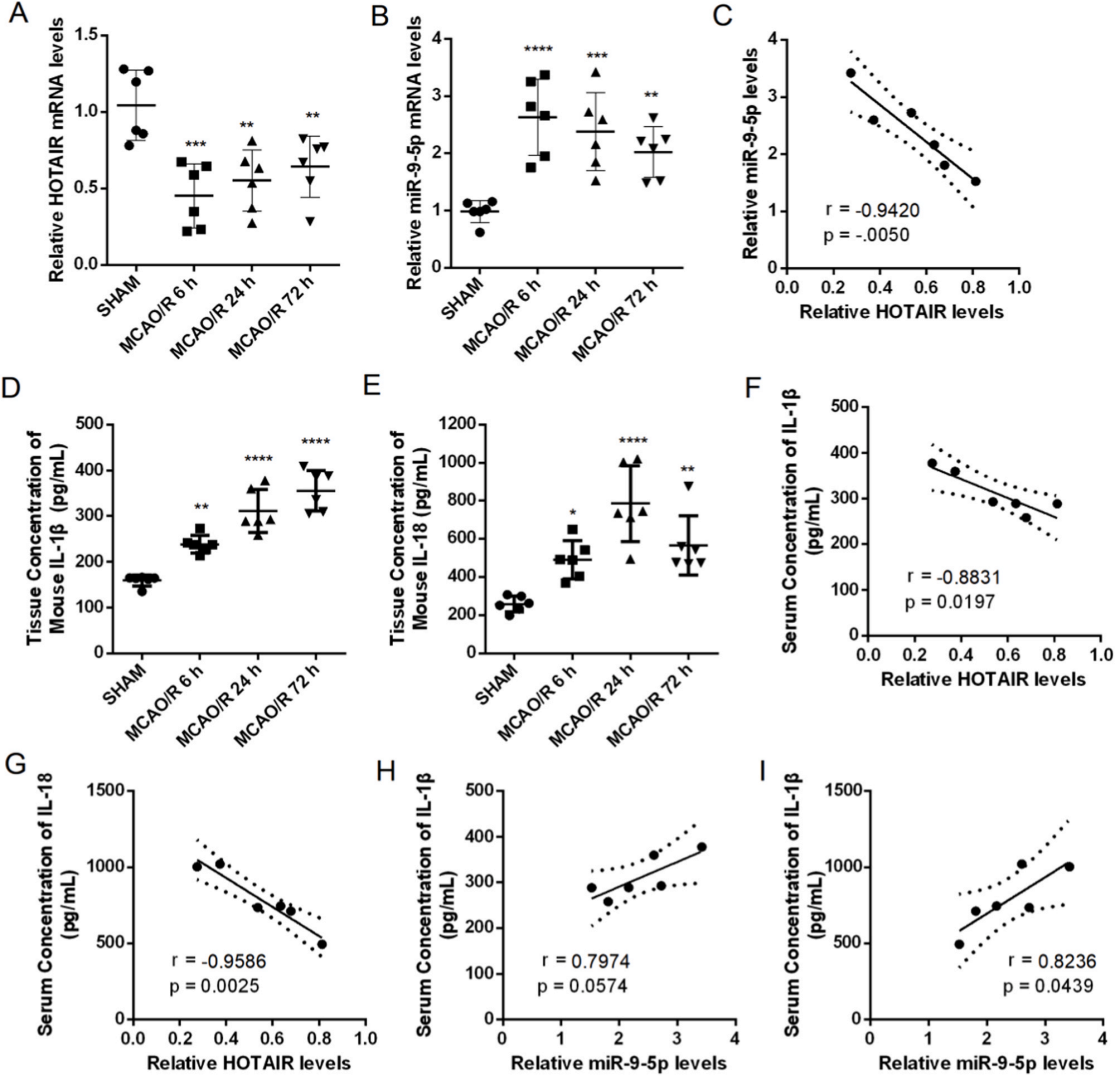
The expression pattern of lncRNA HOTAIR and miR−9–5p, and their association with inflammation response in the MCAO/R mouse model. (A-B) qRT-PCR was used to detect the expression of HOTAIR and miR−9–5p in the brain tissue of mice at 6 h, 24 h, and 3 days after MCAO/R. (C) Pearson’s correlation analysis between the expression of HOTAIR and miR−9–5p at 24 h after MCAO/R. (D-E) Quantitative analysis of IL-1β and IL-18 in brain tissue of MCAO/R mice was measured by ELISA. (F-I) Pearson’s correlation analysis between the expression of HOTAIR/miR−9–5p and IL-1β/IL-18 at 24 h after MCAO/R. r, correlation coefficients. *N* = 6 per group. Data are expressed as mean ± SD. **P < 0.01, ***P < 0.001, ****P < 0.0001 versus the SHAM group.

To validate the role of the HOTAIR-miR−9–5p axis in the inflammatory response during ischemia-reperfusion in animal models, an MCAO/R mouse model was established. The expression of HOTAIR and miR−9–5p in the brain tissue of MCAO/R mice was detected at 6 h, 24 h, and 72 h after MCAO/R. qRT-PCR results showed decreased expression of HOTAIR, while miR−9–5p was increased in the brain tissue at 6 h, 24 h, and 72 h after MCAO/R (Fig. 2A-B), which was consistent with the expression pattern of lncRNA HOTAIR and miR−9–5p in the serum of IS patients. Pearson correlation analysis indicated that HOTAIR was negatively correlated with miR−9–5p (r = −0.9420, *P* < 0.01) (Fig. 2C). Furthermore, ELISA results demonstrated that IL-1β and IL-18 expression levels in brain tissue were significantly elevated in the MCAO/R group compared to the sham group at both 24 h and 72 h post-reperfusion (Fig. 2D-E). Pearson’s correlation analysis revealed that the expression levels of IL-1β and IL-18 were positively correlated with miR−9–5p expression in brain tissue of MCAO/R, and negatively correlated with HOTAIR expression (Fig. 2F-I). These findings collectively suggest that the HOTAIR-miR−9–5p axis may contribute to the inflammatory response observed in MCAO/R mice.

### 3.3 Increased inflammatory response and downregulated FOXP1 expression in the brain of MCAO/R mice

**Fig. 3:**
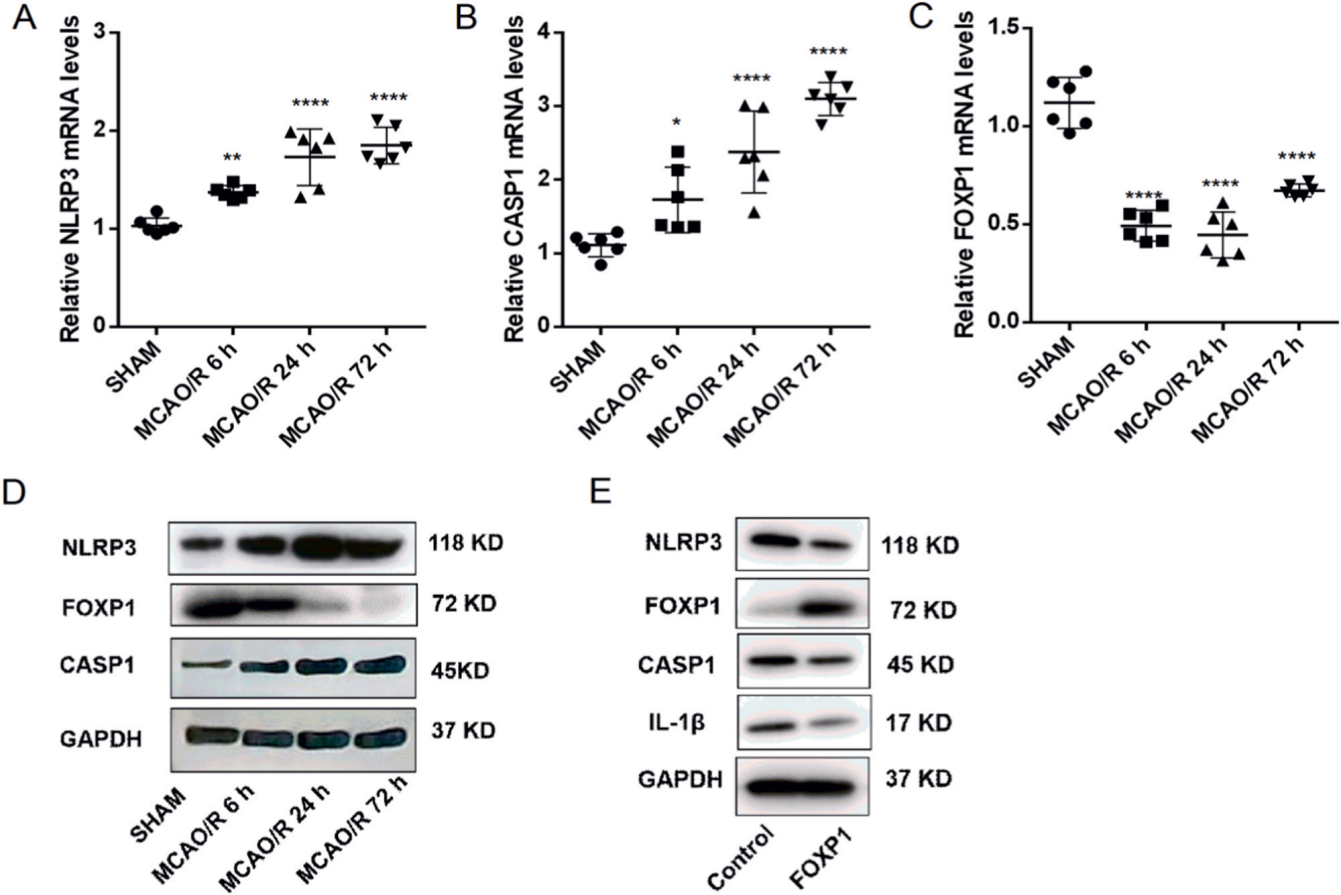
The inflammatory response in the brain of MCAO/R mice was increased while FOXP1 was decreased. (A-C) qRT-PCR was used to detect the mRNA expression of caspase1, NLRP3 and FOXP1 in the brain tissue of mice at 6 h, 24 h, and 3 days after MCAO/R. (D) Western blotting was used to detect protein levels of NLRP3, CASP1 and FOXP1 in mouse brain tissue at different time points after MCAO/R. *N* = 6 per group. (E) The SH-SY5Y cells were harvested at 48 h post-transfection with an overexpression plasmid encoding FOXP1, and then western blotting was employed to quantify the protein levels of IL-1β, CASP1, NLRP3 and FOXP1, using GAPDH as the normalization control. *N* = 3 independent experiments. Data are expressed as mean ± SD. *P < 0.05, **P < 0.01, ****P < 0.0001 versus the SHAM group.

Given the marked upregulation of IL-1β and IL-18 expression in the brain tissues of MCAO/R mice, the expression levels of inflammasome-related proteins were investigated using qRT-PCR and Western blotting. Our findings demonstrated a marked elevation in the expression of NLRP3 and CASP1 within the MCAO/R group, as illustrated in Fig. 3A, B, and D. Furthermore, a significant reduction was observed in both the mRNA and protein levels of FOXP1, a factor known to inhibit NLRP3 inflammasome activation, in the brain tissues of MCAO mice, as shown in Fig. 3C and D.

To explore the effect of FOXP1 on NLRP3 inflammasome activation, gain-of-function experiments were conducted by transfecting SH-SY5Y cells with a FOXP1 overexpression vector (pc-FOXP1). The results indicated that pc-FOXP1 could suppress NLRP3 expression, suggesting an inhibitory effect on NLRP3 inflammasome activation, as depicted in Fig. 3E.

### 3.4 The expression of HOTAIR and FOXP1 was negatively regulated by miR−9–5p

**Fig. 4:**
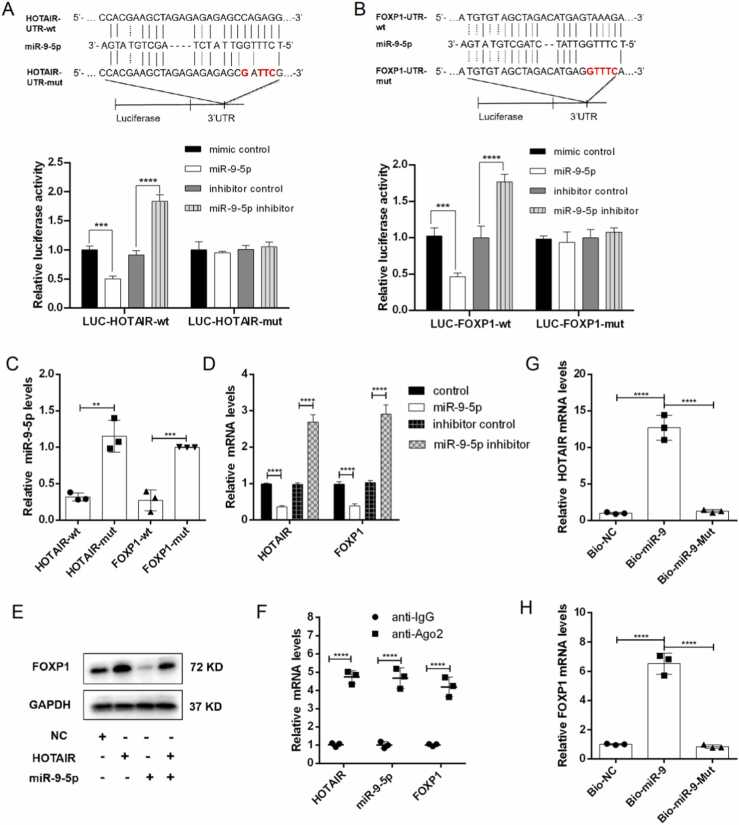
HOTAIR and FOXP1 as direct targets of miR−9–5p. (A, B) The predicted 3’ untranslated region (3’UTR) sequences of HOTAIR and FOXP1, encompassing the miR−9–5p binding sites, were identified using bioinformatics analysis. Subsequently, dual luciferase reporter assays were performed in SH-SY5Y cells to validate the targeting interactions between HOTAIR and miR−9–5p, as well as between miR−9–5p and FOXP1. (C) The expression of miR−9–5p was tested by qRT-PCR in SH-SY5Y cells transfected with HOTAIR-wt/mut or FOXP1-wt/mut. (D) The mRNA levels of HOTAIR and FOXP1 in SH-SY5Y cells transfected with miR−9–5p mimic or inhibitor were determined by qRT-PCR. (E) The protein expression of FOXP1 in SH-SY5Y cells transfected with miR−9–5p mimic or HOTAIR was determined by western blotting. (F) RIP assay assessed the binding of HOTAIR/miR−9–5p/FOXP1 and Ago2. (G-H) Pulldown assay was applied to validate the interaction between HOTAIR and miR−9–5p, as well as between FOXP1 and miR−9–5p. Data are expressed as mean ± SD. *N* = 3 independent experiments. Statistical significance: **P < 0.01, ***P < 0.001, ****P < 0.0001.

Mounting evidence has shown that lncRNAs regulate biological activity through the lncRNA-miRNA-mRNA regulatory network. Therefore, we sought to explore the potential targeting relationships among HOTAIR, miR−9–5p, and FoxP1. Using miRanda software, miR−9–5p was found to target HOTAIR and FoxP1 (Fig. 4A-B). To verify the targeting relationship, a dual-luciferase reporter assay was conducted in SH-SY5Y neurons, and the results substantiated that miR−9–5p significantly mitigated the luciferase activity of LUC-HOTAIR-wt and LUC-FoxP1-wt, while yielding little effect on that of the LUC-HOTAIR-mut and LUC- FoxP1-mut (Fig. 4A-B). Furthermore, miR−9–5p expression was markedly lower in cells transfected with wild-type HOTAIR/FOXP1 compared to their mutant-transfected counterparts (Fig. 4C). The miR−9–5p mimic reduced HOTAIR and FOXP1 expression, whereas the miR−9–5p inhibitor increased their expression (Fig. 4D).

Remarkably, Western blot experiments showed that FOXP1 protein expression was downregulated by miR−9–5p transfection but upregulated by HOTAIR transfection. Crucially, this HOTAIR-driven FOXP1 upregulation was completely reversed by co-transfection with miR−9–5p, confirming their antagonistic interaction (Fig. 4E). RIP results indicated that HOTAIR, FoxP1 and miR−9–5p were preferentially enriched in Ago2-containing beads compared to those harboring control immunoglobulin G (IgG) antibody (Fig. 4F). Biotinylated RNA pull-down experiments revealed selective enrichment of HOTAIR (Fig. 4G) and FOXP1 (Fig. 4H) only when using wild-type miR−9–5p probes, but not with sequence-mutated oligos. This specificity confirms direct targeting of HOTAIR and FOXP1 by miR−9–5p, leading to their downregulation. Taken together with previous findings, these results demonstrate that miR−9–5p directly targets and downregulates both HOTAIR and FOXP1.

### 3.5 HOTAIR regulates NLRP3 expression via the miR−9/FOXP1 pathway

**Fig. 5:**
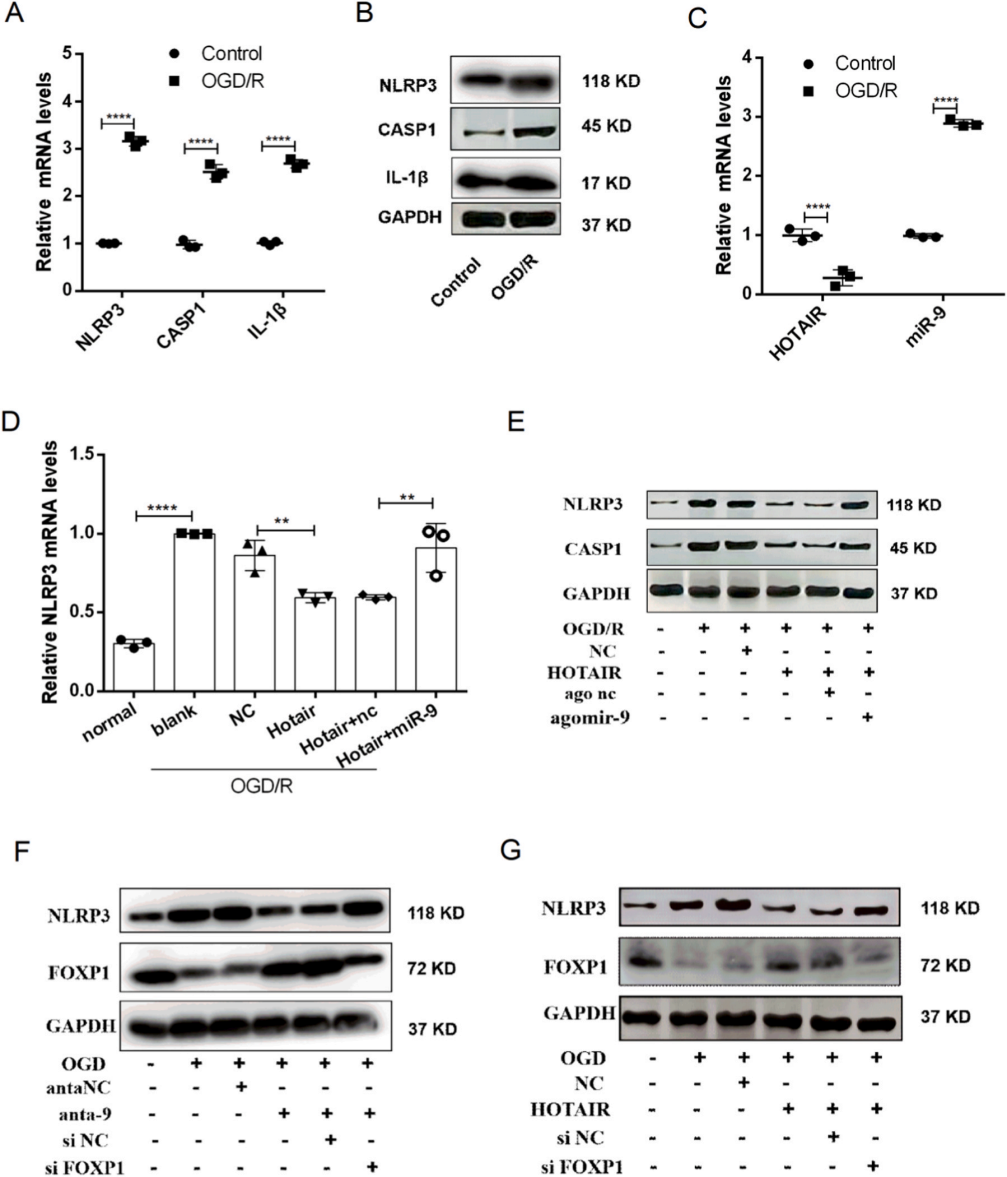
HOTAIR modulates NLRP3 expression via the miR−9/FOXP1 regulatory axis in OGD/R-treated cells. (A-B) qRT-PCR and western blotting were employed to quantify the mRNA and protein levels of NLRP3, CASP1 and IL-1β in SH-SY5Y cells subjected to OGD/R treatment. (C-D) The mRNA expression levels of HOTAIR, miR−9–5p and NLRP3 were determined by qRT-PCR. (E-G) Western blotting was employed to compare the expression of NLRP3, CASP1 and FOXP1 in SH-SY5Y cells following OGD/R treatment. *N* = 3 independent experiments. Data are expressed as mean ± SD. Statistical significance: **P < 0.01, ***P < 0.001, ****P < 0.0001.

In SH-SY5Y cells subjected to a 4-hour OGD/R challenge, qRT-PCR and western blotting analyses revealed a marked elevation in the expression levels of NLRP3, caspase−1, IL-1β, and IL-18 in OGD/R-exposed cells (Fig. 5A-B). Concurrently, HOTAIR expression was significantly downregulated, whereas miR−9–5p levels were elevated in SH-SY5Y cells subjected to OGD (Fig. 5C). These findings were consistent with observations in patients with IS as well as in MCAO/R mouse models.

In the OGD/R cell model, overexpression of HOTAIR attenuated the expression of NLRP3 and CASP1, whereas a miR−9–5p inhibitor could counteract the effect (Fig. 5D-E). Analogously, inhibition of miR−9–5p resulted in a reduction of NLRP3 expression through modulation of FOXP1 (Fig. 5F). Moreover, HOTAIR overexpression suppressed NLRP3 expression by regulating FOXP1 (Fig. 5G). Collectively, these results provide compelling evidence indicating that HOTAIR inhibits NLRP3 expression through the miR−9/FOXP1 regulatory pathway.

### 3.6 Silencing miR−9–5p protects against cerebral ischemia/reperfusion injury and suppresses NLRP3 inflammasome activation

**Fig. 6:**
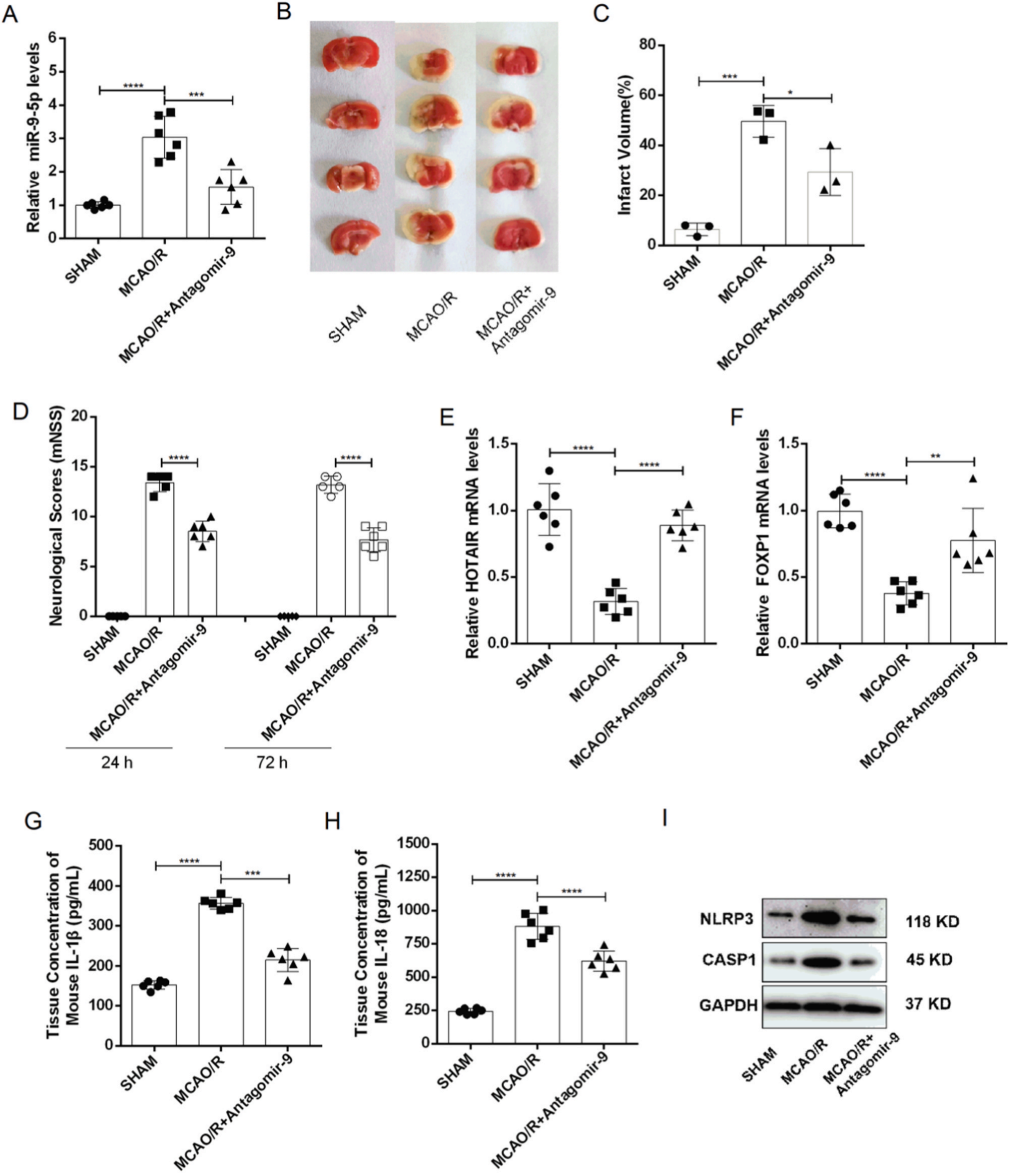
Attenuation of miR−9–5p ameliorates ischemia/reperfusion injury and relieves NLRP3 inflammasome activation. (A) Antagomir−9 or its negative control (antaNC) was administered to MCAO/R mice via intracerebroventricular injection. Subsequently, miR−9–5p expression levels were assessed using qRT-PCR. *N* = 6 per group. (B-C) Representative TTC-stained brain tissue sections are shown, along with a quantitative analysis of cerebral infarction volume in mice. *N* = 3 per group. (D) Neurological deficit scores were evaluated using mNSS after I/R. (E-F) qRT-PCR was employed to quantify the mRNA expression of HOTAIR and FOXP1 in the treated MCAO/R mice. (G-H) The quantitative analysis of IL-1β and IL-18 in the brain tissue of MCAO/R mice was performed using ELISA. (I) Western blotting was utilized to compare the protein expression of NLRP3 and CASP1 in the treated MCAO/R mice. *N* = 6 per group. Data are expressed as mean ± SD. Statistical significance: *P < 0.05, **P < 0.01, ***P < 0.001, ****P < 0.0001.

To evaluate the effect of miR−9–5p on cerebral I/R injury *in vivo*, antagomir−9–5p or its negative control, antagomir-NC, was administered via intracortical injection into the mouse cortex. After a 48-hour interval, the mice underwent 1 h of MCAO, followed by 24 h of reperfusion. Neurological deficits and infarct volume were assessed 24 h post-reperfusion. As depicted in Fig. 6A-C, treatment with antagomir−9–5p significantly reduced infarct volume and neurological deficits, indicating that silencing miR−9–5p mitigates MCAO/R-induced injury *in vivo*. qRT-PCR analysis demonstrated that MCAO/R significantly downregulated the expression of HOTAIR and FOXP1 while concurrently upregulating miR−9–5p levels (Fig. 5D-F). However, these alterations were reversed following antagomir−9–5p treatment. Furthermore, ELISA and Western blotting analyses revealed that silencing miR−9–5p could suppress NLRP3 inflammasome activation (Fig. 6G-I).

## 4 Discussion

Currently, severe CIRI is frequently encountered in clinical settings, leading to enduring neurological impairments in patients (Gao et al., 2024). Numerous factors contribute to CIRI, and at present, there remains a lack of specific therapeutic interventions (Xu et al., 2023). Consequently, it is crucial to gain a comprehensive understanding of the underlying mechanisms of CIRI to develop novel treatment approaches.

Previous studies have reported the involvement of multiple lncRNAs in the progression of AIS. For example, elevated levels of NORAD have been shown to serve as a diagnostic biomarker for AIS and to predict adverse clinical outcomes (Liu et al., 2023). Moreover, LINC00265 expression was notably decreased in AIS patients and was closely linked to disease severity. Functional studies indicate that overexpression of LINC00265 might hinder AIS progression by modulating the miR−155–5p/TRIM32 axis (Lv and Xie, 2025). Current evidence suggests that lncRNA HOTAIR in neonatal hypoxic-ischemic encephalopathy mediates OGD/R-induced cell injury and angiogenesis in an EZH2-dependent manner (Wang et al., 2022).

Consistently, the present study showed that HOTAIR was significantly reduced in the serum of patients with IS, whereas miR−9–5p levels were significantly increased. Likewise, serum miR−9–5p levels were upregulated in patients with AIS compared with healthy individuals (Wang et al., 2021). Moreover, in our study, the pattern of HOTAIR and miR−9–5p was similarly observed in OGD/R and MCAO/R models, consistent with the literature (Zhou et al., 2020). These findings suggest that HOTAIR and miR−9–5p may serve as potential diagnostic biomarkers for acute ischemic stroke.

Recent research has underscored the crucial role of the NLRP3 inflammasome in post-stroke neurovascular injury, making it a highly promising therapeutic target for ischemic stroke (Sun et al., 2019). Both clinical observations and experimental models have consistently shown significant upregulation of NLRP1/NLRP3 inflammasomes and their downstream effector cytokines, namely IL-1β and IL-18, in peri-infarct areas (Ismael et al., 2018). Pharmacological blockade of the NLRP3/caspase−1 pathway was found to reduce infarct volume and preserve BBB integrity across multiple preclinical stroke models (Chen et al., 2021). Furthermore, NLRP3 activation could exacerbate ischemic injury through additional pathways, including amplification of the IL-23/IL-17 inflammatory axis (Wang et al., 2019).

Studies have shown that inhibition of miR−9–5p downregulates the expression of inflammatory factors, including IL-1β, IL-6, iNOS, and TNF-α (Wang et al., 2023). The present study revealed that silencing miR−9–5p could decrease the expression of IL-1β and IL-18 in MCAO/R mice. While HOTAIR knockdown via shRNA (sh-HOTAIR) led to a marked reduction in neuronal pyroptosis, HOTAIR overexpression suppressed propofol-induced neuronal pyroptosis by regulating the miR−455–3p/ NLRP1 axis (Gong et al., 2021), suggesting that the roles of HOTAIR may vary under different physiological and pathological conditions. Our investigation also demonstrated decreased HOTAIR levels in IS patients and MCAO/R mice, along with an increased NLRP3 inflammatory response (as indicated by increased expression of IL-1β and IL-18). A negative correlation was identified between HOTAIR expression and the inflammatory response. Moreover, the correlation between miR−9–5p and the inflammatory response was positive. However, the specific mechanisms underlying the HOTAIR-miR−9–5p-NLRP3 axis remain to be further studied.

LncRNAs can serve as miRNA hosts, competitively binding with miRNAs to mRNAs (Xue et al., 2022). Moreover, they can either directly bind to miRNAs to downregulate them or indirectly modulate them via mediators (Braga et al., 2020). As reported, HOTAIR acted as a competing endogenous RNA (ceRNA) to sequester miR−195–5p and elevate ABCG2 expression, leading to oxaliplatin resistance in GC cells (Luo et al., 2023). Our database analysis identified miR−9–5p as a potential target of HOTAIR and Forkhead Box P1 (FOXP1), which is a vital regulator of IS progression (Li et al., 2023). The binding of miR−9–5p to HOTAIR or FOXP1 was confirmed by dual-luciferase, RIP, and RNA pulldown experiments, revealing a novel HOTAIR/miR−9–5p/FOXP1 axis in the IS progression. However, the relationship between this axis and the NLRP3 inflammatory response remains to be elucidated.

FOXP1 was reported to suppress NLRP3 activation in atherosclerosis (Zhuang et al., 2019). Current evidence suggests that FOXP1 can prevent brain damage during cerebral I/R injury by suppressing NLRP3 inflammasome activation, whereas FOXP1 knockdown yields contrasting effects (Li et al., 2023). In our study, FOXP1 expression in the brains of MCAO/R mice decreased, whereas the NLRP3 inflammatory response increased.

Moreover, under OGD/R stimulation, HOTAIR expression was notably downregulated, whereas miR−9–5p levels were elevated in SH-SY5Y cells. OGD/R stimulation activated the NLRP3 inflammasome, and this activation was abrogated after HOTAIR overexpression. However, this suppressive effect was reversed by treatment with a miR−9–5p inhibitor. Notably, under OGD/R stimulation, the augmented expression of NLRP3 in SH-SY5Y cells after miR−9–5p inhibition was reversed following FOXP1 knockdown. Similarly, HOTAIR overexpression also suppressed NLRP3 expression by regulating FOXP1. Building on these findings, HOTAIR may function as a ceRNA, elevating FOXP1 expression by sponging miR−9–5p, thereby suppressing NLRP3 expression.

Several limitations should be acknowledged in this study. First, the small clinical sample size limits statistical power and generalizability. While this constraint stems from stringent inclusion criteria designed to ensure data homogeneity and minimize confounders, the results should be considered preliminary. Larger, multi-center cohorts are required to validate the clinical value of the identified axis. Second, although this study preliminarily elucidated the HOTAIR/miR−9–5p/FOXP1/NLRP3 axis in CIRI, the precise mode of action of this mechanism requires further exploration. Third, using SH-SY5Y cells as the sole *in vitro* model presents limitations due to their neuroblastoma origin, characterized by genomic instability and oncogenic metabolic shifts. Nevertheless, compared to primary neurons and other brain cell lines, SH-SY5Y cells offer a human-derived model that is sensitive to pathological stimuli, readily activates NLRP3, and is amenable to culture and genetic manipulation, making them suitable for initial investigations into neuroinflammatory mechanisms. Therefore, findings derived solely from this line should be interpreted with caution regarding physiological relevance to the adult brain. Indeed, validation in primary neurons or complex co-culture systems is essential to confirm translational applicability. Addressing these limitations will require further mechanistic and translational research. Broad-scale clinical trials and prospective observational studies are warranted to rigorously evaluate the therapeutic efficacy and safety profiles of HOTAIR and miR−9–5p in patients with ischemic stroke.

In summary, this research revealed that the differentially expressed lncRNA HOTAIR regulates the NLRP3 inflammatory response in ischemic stroke through the miR−9/FOXP1 axis, functioning as a ceRNA. In addition, inhibition of miR−9–5p could reduce infarct volume by mitigating the NLRP3 inflammatory response in MCAO mice, providing a theoretical basis for the application of non-coding RNAs in the diagnosis and treatment of ischemic stroke.

## CRediT authorship contribution statement

**Shanxin Peng:** Writing – review & editing, Writing – original draft, Project administration, Funding acquisition, Formal analysis, Conceptualization. **Xiaosong Zhu:** Resources, Funding acquisition, Formal analysis, Data curation. **Fengjuan Zhuo:** Resources, Formal analysis. **Tingting Liu:** Writing – original draft, Investigation, Funding acquisition, Formal analysis, Data curation.

## Compliance with ethical standards

All experiments were approved by the Science and Technology Ethics Committee of Linyi People's Hospital of Shandong Second Medical University (approval No.202411-H−057). The study also conformed to the Declaration of Helsinki. Prior to conducting this study, written informed consent was obtained from all participants. The animal experiments were conducted with the approval of the Animal Ethics Committee of Linyi People's Hospital (approval No.202411-A−005) and in strict accordance with the recommendations of the Guide for the Care and Use of Laboratory Animals, published by the US National Institutes of Health. Adequate measures were taken to minimize animal pain and distress.

## Ethics approval and consent to participate

The study was approved by the Science and Technology Ethics Committee of Linyi People's Hospital of Shandong Second Medical University, and written informed consent was obtained from each patient.

## Funding

This work was supported by the grants from the 10.13039/501100007129Natural Science Foundation of Shandong Province (Grant No. ZR2020QH114), Key Research and Development Program of Linyi City (Medical Category) (Grant No. 2024YX0024, 2025YX0051 and 2025YX0086), and the Research Project of Shandong Youth Medical Workers Association (Grant No. 2025ND028).

## Declaration of Competing Interest

The authors declare that they have no known competing financial interests or personal relationships that could have appeared to influence the work reported in this paper.

## Data Availability

All raw data of this study are available from the corresponding author by request.
